# Retinal hemangioblastoma in a patient with Von Hippel-Lindau disease: A case report and literature review

**DOI:** 10.3389/fonc.2022.963469

**Published:** 2022-11-02

**Authors:** Yikeng Huang, Weiwen Hu, Xionggao Huang

**Affiliations:** ^1^ Department of Ophthalmology, the First Affiliated Hospital of Hainan Medical University, Haikou, Hainan, China; ^2^ Department of Ophthalmology, Shanghai General Hospital, Shanghai Jiao Tong University School of Medicine, Shanghai, China

**Keywords:** Von Hippel-Lindau disease (VHL), retinal hemangioblastoma (RH), case report, clinical management, vitreoretinal surgery

## Abstract

**Background:**

Retinal hemangioblastoma (RH) is a rare benign tumor and a considerable number of which are caused by Von Hippel-Lindau disease (VHL). Herein, we described a case of VHL-associated RH with retinal detachment who underwent both laser photocoagulation and vitreoretinal surgery and received satisfactory visual recovery. In addition, we reviewed the current diagnosis, genotype-phenotype association, and treatment of VHL-associated RH.

**Case description:**

A 34-year-old woman presented with vision loss in the right eye at our hospital. Fundus photography and angiography showed retinal detachment and multiple large hemangiomas in the right eye. The visual acuity improved significantly after laser photocoagulation and vitreoretinal surgery. Genetic analyses showed a p.Asn78Ser (c.233A>G) heterozygous missense mutation in the VHL gene.

**Conclusion:**

We described a rare case of VHL-associated RH and may provide a new perspective towards diagnosis and treatment of this disease. RH is one of the most common manifestations of VHL and poses a serious threat to vision. Ophthalmic examination methods include fundus examination and fundus photography, etc. The management of the disease emphasizes timely follow-up, early detection of the lesion, and the decision of treatment options according to the size, location and complications of the lesion, including ablation therapy and vitreoretinal surgery. Clinicians should strengthen the understanding of this rare disease for early detection and treatment.

## Introduction

Retinal hemangioblastoma (RH, also appearing in the literature as retinal capillary hemangioma, retinal capillary hemangioblastoma, or retinal angioma) is a rare benign tumor typically manifested by retinal vascular neoplasms with pink or orange color, nodular appearance, dilated and tortuous feeding and draining blood vessels, as well as exudation involving both perilesional retina and the macula (1–4). It has been reported that a considerable number of RH cases were caused by von Hippel-Lindau disease (VHL), while the rest seemed to be sporadic (1). VHL is a rare autosomal-dominant inherited tumor syndrome that has significant phenotypic heterogeneity and age-related genetic penetrance. With an incidence of approximately 1:36,000, VHL is believed to be one of the most common hereditary tumor syndromes that has various clinical manifestations mainly including RH, central nervous system hemangioblastoma(CNSH), renal cell carcinoma(RCC), pheochromocytoma (PCC) and pancreatic cysts etc (3, 5, 6). It is important to note that RH is the most common and probably the only presentation in VHL patients and is the initial manifestation in up to 77% of them (1, 4–6). The penetrance of RH would reach up to 90% in VHL patients over 60 years (7, 8), and about half of the cases present with multiple and bilateral RHs, which poses a serious threat to the patient’s vision (6). As an ophthalmologist, identifying RH and determining whether it is associated with VHL is extremely important for the early diagnosis and treatment of this rare hereditary syndrome that may occur in these patients and their families.

Herein, we described a rare case of VHL-associated RH with severe visual impairment due to retinal detachment. Laser photocoagulation and vitreoretinal surgery were performed, and her vision recovered satisfactorily. In addition, we provided a literature review of VHL-associated RH to summarize the current diagnosis, genotype-phenotype association, and treatment of this disease. Our study may provide a new perspective towards ophthalmic diagnosis and treatment of VHL-associated RH.

## Case report

**Figure 1:**
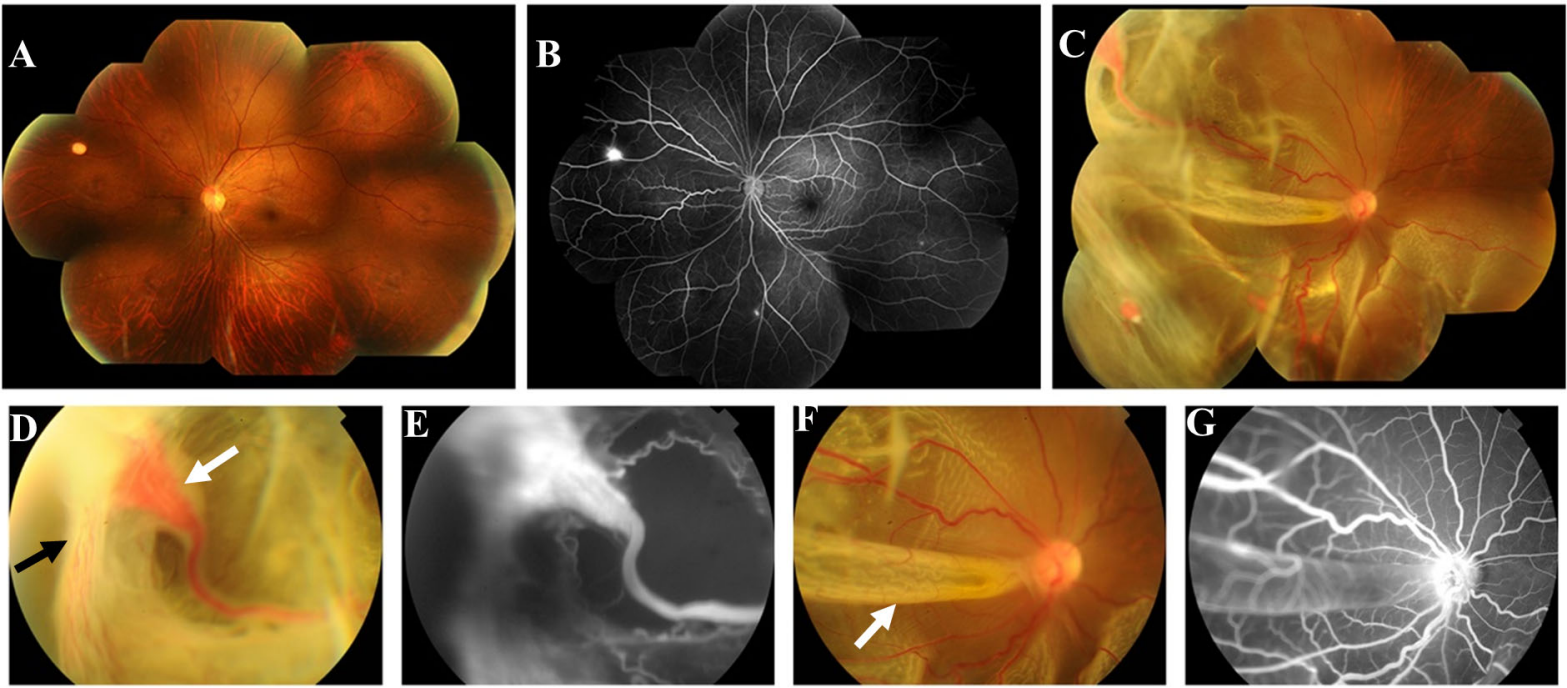
First fundus photograph and fluorescence angiography (FA) of the patient. Fundus photography of the left eye **(A)** revealed a suspected hemangioma at 10 o ‘clock of the peripheral retina. FA of the left eye **(B)** indicated that there were multiple RH lesions in the peripheral retina with local hyper-fluorescence, which were not shown by fundus photography. Fundus photograph of the right eye **(C, D, F)** revealed 4 orange-red hemangiomas in the superior and inferior temporal periphery retina with dilated and tortuous feeding and draining blood vessels, and the largest of which was about 3.75 x 2.25mm(white arrow in **(D)** with neovascular membranes in its temporal side (black arrow in **(D)**. Retinal detachment could be seen from 3 o ‘clock to 11 o ‘clock with retinal fold formation(white arrow in **(F)**, involving the posterior pole of the eyeball. FA of the right eye **(E, G)** showed hyper-fluorescence and fluorescence leakage at the lesion, accompanied by a surrounding non perfusion area.

A 34-year-old woman was admitted to the hospital with vision loss in the right eye over the last 3 days. Upon initial ophthalmological examination, the patient’s visual acuity was HM/BE 20cm OD with no improvement in corrected vision and 1.0(Snellen chart)OS with corrected vision improving to 1.0(+1.0DS/-2.0DC*10), and intraocular pressure was 17 mmHg OD and 20 mmHg OS. Nothing noteworthy was observed in the anterior segment of either eye and observations of the ocular fundus showed no abnormalities in the left eye. However, from 3 o ‘clock to 11 o ‘clock in the fundus of the right eye, the retina showed a state of grey-white bulge and flutter without retinal holes, and the macula was involved. Fundus photography further showed retinal detachment with multiple large hemangiomas in the right eye and a suspected hemangioma in the left eye (**Figure 1**). There were 4 orange-red hemangiomas in the superior temporal periphery and inferior temporal detachment area of the retina in the right eye with dilated and tortuous feeding and draining blood vessels as well as evident peripheral neovascular membranes. Fluorescence angiography(FA) showed early hyper-fluorescence with late leakage. Optical coherence tomography(OCT) showed retinal detachment. Prior to this visit, the patient had undergone head CT examination and found an intracranial hemangioma (**Supplementary Figure 1**), which was subsequently treated surgically at another hospital. Abdominal ultrasonography was also performed and showed multiple cysts present in the pancreas and right kidney while spinal CT showed no abnormalities. She was diagnosed with type 2 diabetes at the age of 32 and the HbA1c level on admission was 14.2%. Her mother suffered from diabetes and died of unknown causes at the age of 31. Her sister also underwent surgery for intracranial hemangioma. Although the patient’s mother and sister were not conclusively diagnosed with VHL by clinical or genetic testing, we highly suspect them to have the disease based on the available clinical results. Moreover, we did ophthalmologic examinations for the patient’s family who were willing to be examined and the results were shown in the supplementary materials (**Supplementary Figure 2**).

**Figure 2:**
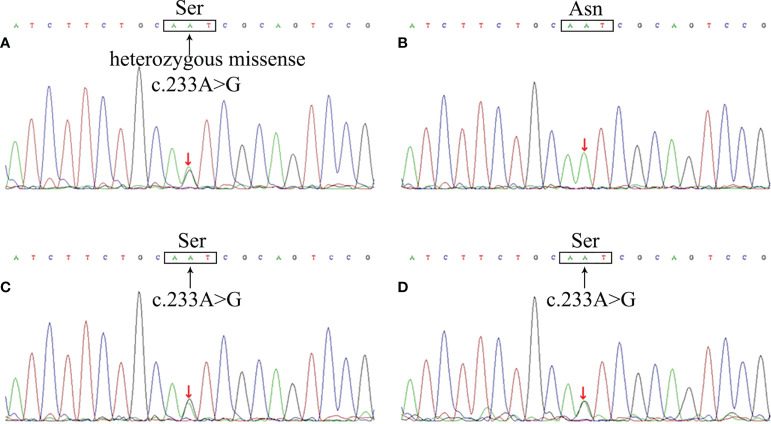
The VHL gene mutation of the patient and her family members. Sequencing analysis revealed a c.233A>G heterozygous missense mutation in VHL gene of the patient **(A)**, resulting in the substitution of Ser from Asn in the 78th amino acid site of VHL protein. The patient’s daughter **(C)** and son **(D)** showed the same mutation, whereas her husband **(B)** did not.

Based on family history, clinical manifestations and imaging, we suspected that the patient had VHL. To further confirm this, the patient and her family underwent genetic sequencing and the results showed a c.233A>G heterozygous missense mutation (amino acid p.Asn78Ser) in exon 1 of the VHL gene of the patient, her daughter and her son (**Figure 2**), and the genogram of the family was shown in the supplementary materials (**Supplementary Figure 3**). The patient was definitely diagnosed with VHL based on these findings.

**Figure 3:**
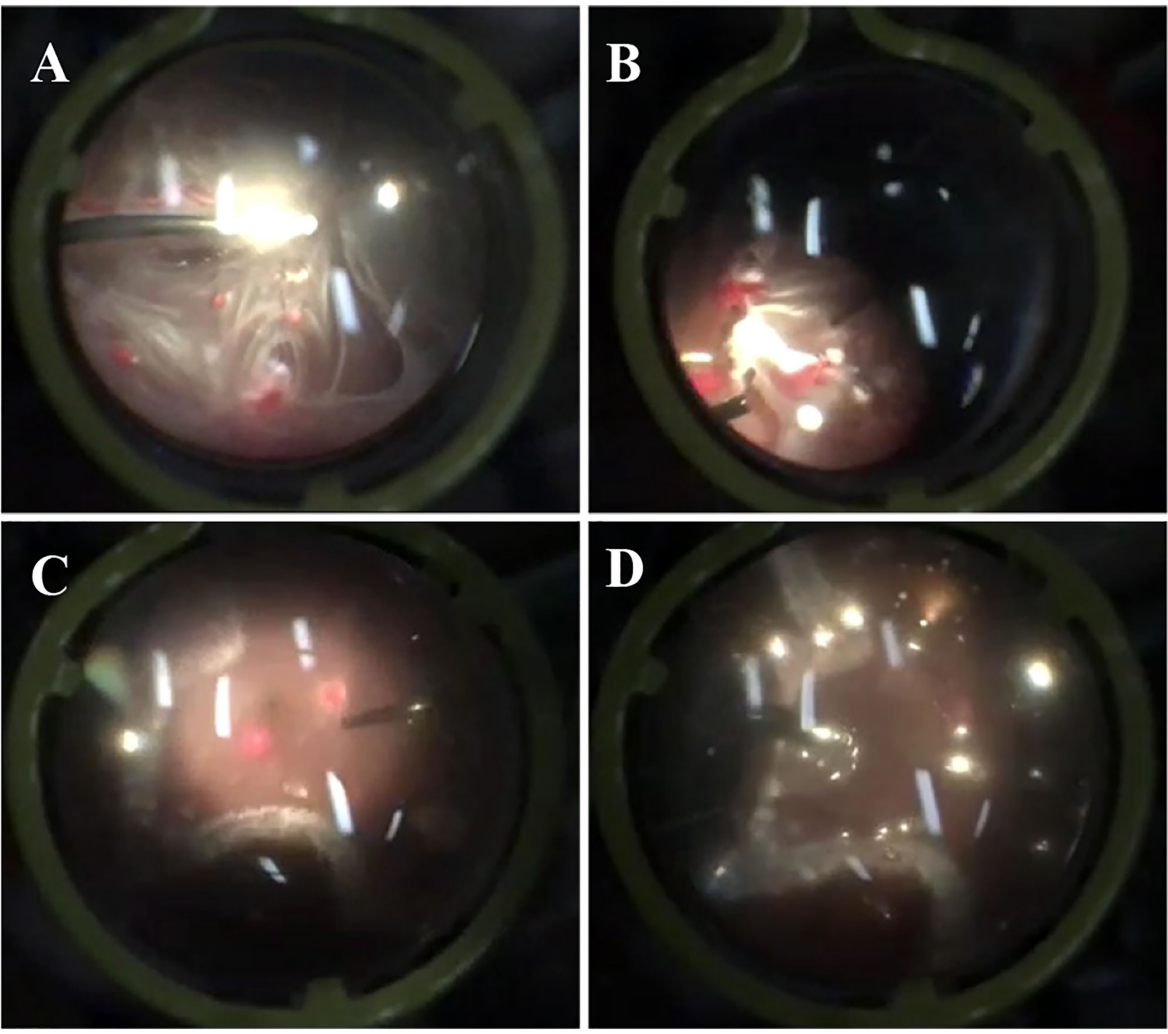
Screenshots of the surgery for the patient’s right eye. The above screenshots mainly showed the process of pars plana vitrectomy **(A)**, lesion resection **(B)**, endolaser photocoagulation **(C)** and silicone oil tamponade **(D)**.

**Figure 4:**
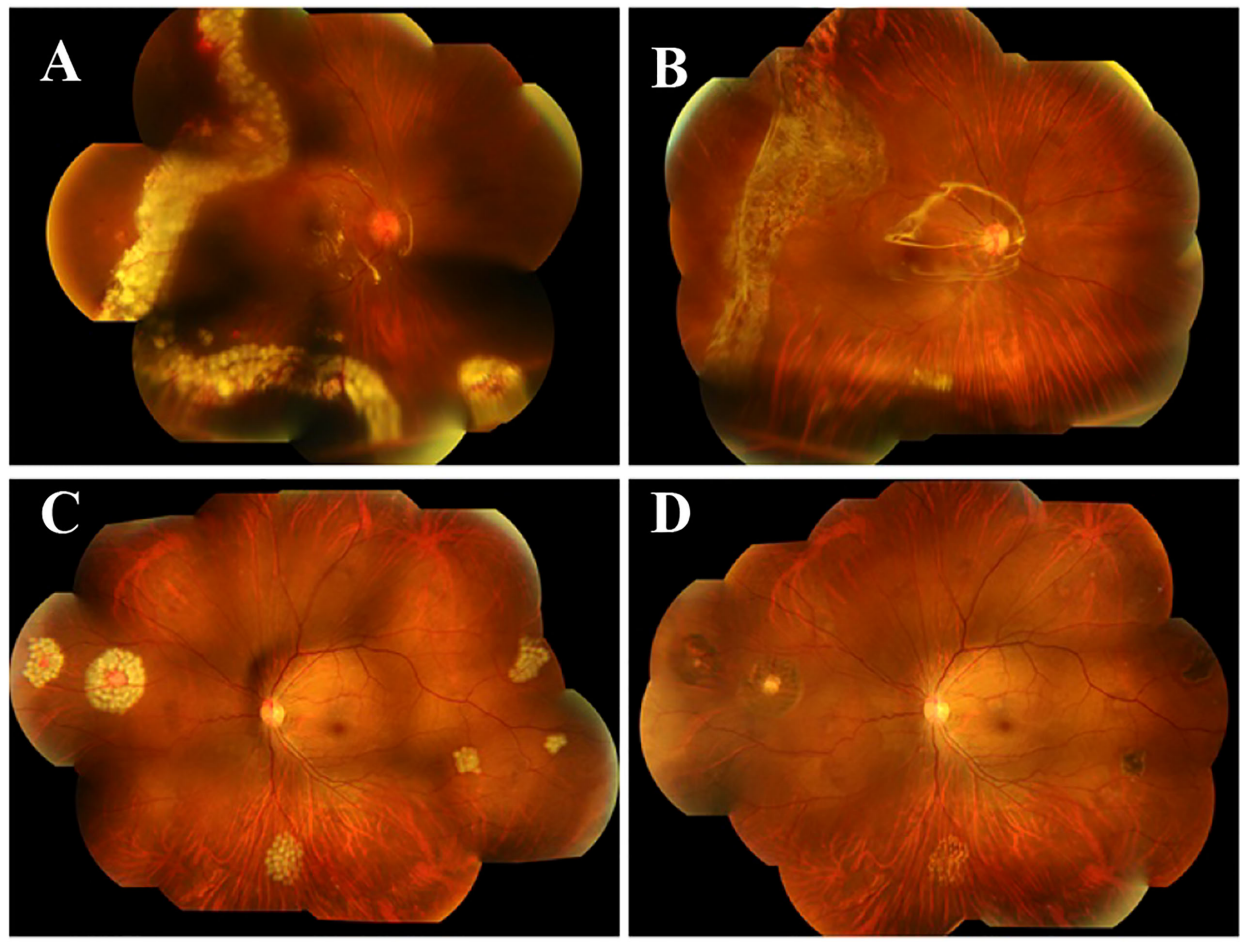
Fundus photography one day and 3 months after the surgery. Fundus photography one day after the surgery showed that the retinal detachment of the right eye recovered **(A)** and the hemangiomas of the left eye scarred **(C)** following the treatment. Fundus photography 3 months after the surgery **(B, D)** showed good fundus recovery in the both eyes.

The patient then underwent pars plana vitrectomy(PPV), lesion resection, endolaser photocoagulation and silicone oil tamponade for the right eye. During the operation, after a 23-gauge(23-G) PPV the four peripheral hemangiomas and the neovascular membranes were extracted using forceps, and an endolaser photocoagulation (argon ion laser, 532nm) was applied around the lesions to repair retinal detachment (**Figure 3**). As for the left eye, laser photocoagulation treatment(argon ion laser) was performed directly to the hemangioma and the surrounding area in order to induce degeneration of the tumor and its feeding and draining vessels. The retinal detachment of the right eye recovered and the hemangiomas of the left eye scarred following the treatment (**Figure 4**). At a follow-up examination before silicone oil extraction, 3 months after the surgery, the patient’s visual acuity had improved to 0.05 OD with corrected vision improving to 0.6(+4.50DS/+1.25DC*10) and 0.6 OS with corrected vision improving to 1.0(+0.25DS/-2.50DC*15). A timeline figure of the diagnosis and treatment of this case was provided in the supplementary material (**Supplementary Figure 4**).

For the patient’s children, both of her daughter and son present the same heterozygous pathogenic mutation in the VHL gene, and her son (12 years old) had already had multiple retinal hemangiomas. We performed laser photocoagulation treatment for the patient’s son and suggested further cranial and abdominal examinations, but the patient refused. Follow-up examinations showed ideal recovery in his both eyes. However, it was with regret that we were informed of his death due to a cerebral hemorrhage 2 years after the treatment.

## Discussion

### Pathogenesis, diagnosis and clinical manifestations

VHL is a rare hereditary tumor syndrome with RH as one of its most common and first clinical manifestations (5, 7, 9). Biallelic inactivation of VHL gene, a known tumor suppressor gene, is considered to be the pathogenesis of VHL in accordance with Knudson’s two-hit hypothesis of tumorigenesis (3, 5, 6, 9, 10). In most cases, VHL patients receive one germline mutant allele from their parents and then acquire another allele for somatic mutation through deletion, insertion, missense, truncation mutation or promoter hypermethylation. As a result, the VHL protein(pVHL) may be lost or inactivated resulting in changes of various cellular functions in both hypoxia-inducible factor(HIF) dependent and independent way (5, 10). In fact, the most critical function of pVHL is its adaptive response to hypoxia conditions, which is achieved through its interaction with HIF. Widely expressed in cells throughout the body, pVHL interacts with elongin B, C and Cullin-2 through one of its two protein-binding domains, α-domain to form a complex that acts as an E3 ubiquitin ligase (11–13). Under normoxic conditions, the complex recognizes and mediates ubiquitination degradation of the HIF-α subunit through the β domain of pVHL, whereas in anoxic conditions, the HIF-α subunit remains stable and binds to HIF-β subunit to activate a large number of target genes that mediate the regulation of different processes such as angiogenesis(VEGF, PDGF, CTGF), proliferation(TGFα), apoptosis(CyclinD1), and metabolism(GLUT-1, 6-PFK, PDK) (3–6, 9, 10, 14). Therefore, the loss or inactivation of pVHL produces a similar effect to that in anoxic environment and eventually leads to multiple tumorigenesis.

The current clinical diagnostic criteria for VHL are as follows: ① one typical VHL tumor(including RH, CNSH, RCC, PCC or pancreatic neoplasm etc.)with a clear family history of VHL;②two hemangioblastomas(RH or CNSH) or one hemangioblastoma and a visceral tumor(PCC, RCC or pancreatic neoplasm etc.) (3, 6, 10, 15). Although VHL patients with typical lesions can be diagnosed based on clinical diagnostic criteria, there are still many suspected cases with atypical lesions that require genetic testing for further confirmation or exclusion (3, 5, 15). However, genetic testing is not a panacea as it was reported that the detection rate of VHL gene mutations were much lower in patients who lacked typical clinical manifestations(24%) than in classic cases with typical features(95%) (16). Therefore, recognizing that no single diagnostic method is enough to completely avoid negative results, it is very important to combine the patient’s clinical manifestations and the results of various tests, including genetic tests.

As RH is the main feature of VHL in the eye, identification of VHL-associated RH is crucial for ophthalmologists to establish early diagnosis and provide further screening and treatment. Under the ophthalmoscope, RH usually presents as pink or orange nearly round lesions with obvious dilated and tortuous feeding and draining blood vessels around the tumor in most cases. Secondary exudation, proliferation, hemorrhage, and even exudative or tractive retinal detachment may occur resulting in visual impairment in some cases (2–6, 9, 10, 17). According to the distribution of the lesions in the retina, RH can be divided into peripheral RH and juxtapapillary RH. The former is considered to be the most common ocular lesion of VHL, which is usually located in the superior or inferior temporal region of the peripheral retina. The latter occurs on the optic nerve head or within the juxtapapillary region. Although less common than peripheral RH, it causes more serious damage to vision and limits treatment due to its special location and higher possibility of retinal detachment and macular exudation (9, 18, 19). High resolution fundus photography and FA can improve the detection rate of RH that are difficult to find under the ophthalmoscope. FA often shows marked early hyper-fluorescence with late leakage. For advanced lesions, ocular ultrasound and OCT can be helpful in assessing the extent of macular exudation and retinal detachment (3, 5, 9, 20). Although diagnosis of RH is generally not difficult based on its characteristics, in some cases it should be distinguished from other retinal vascular tumors such as retinal cavernous hemangioma, Wyburn-Mason syndrome, and retinal vasoproliferative tumor (9).

In our case, the patient mainly presented with significant visual impairment of the right eye caused by retinal detachment, and fundus photography revealed four retinal hemangiomas in the temporal peripheral retina with obvious dilated feeding blood vessels and significant hyperplasia. On this basis, we believed that the patient’s retinal detachment was mainly tractional and caused by secondary hyperplasia of hemangioma, although exudation could not be ruled out. FA further confirmed the location and size of the hemangioma in the right eye, as well as several small lesions that had not been detected by fundus photography in the left eye. Considering bilateral, multifocal onset and severe secondary retinal detachment, the patient’s medical history and family history were carefully examined. Finally, combining the patient’s history of CNSH, multiple cysts in the pancreas and right kidney, and clear family history, we determined the clinical diagnosis of VHL. To further verify the diagnosis, genetic test was performed on the patient and her family, and the results were as described above.

We noticed that both the patient and her mother had diabetes at their early 30s, and considering the patient’s history of pancreatic cysts, we wondered whether the onset of diabetes might be related to pancreatic cysts caused by VHL. B. Mukhopadhyay et al. reported that extensive serous microcystic adenomas of the pancreas in patients with VHL is closely related to diabetes mellitus (21). Yun Hyi Ku et al, reported a case of VHL with multiple renal and pancreatic cysts associated with gestational diabetes mellitus (22). However, more studies have shown that while the majority of VHL patients may have pancreatic tumors, they are mostly asymptomatic and diabetes is rare in these cases (21–23). In fact, animal studies have shown that loss of the VHL gene in islet β cells leads to impaired glucose tolerance in mice, but whether this is associated with pancreatic tumors remains unclear (24, 25). In addition, in our case, the patient’s level of blood glucose was very high(HbA1c 14.2%), but no diabetic retinopathy was observed, which might be related to the short course of the onset of diabetes.

### Genotype-phenotype association

It has been reported that there is significant heterogeneity in the clinical manifestations of VHL within and between families, which may be largely related to the diversity of VHL gene mutations (7, 14).With regard to ocular VHL disease, the germline mutations of VHL gene with the highest correlation with RH are missense mutation, truncation mutation and complete deletion (7, 12, 26, 27). Wai T. Wong et al. reported that patients with complete VHL deletion had a lower incidence of RH and better vision than missense mutation and truncation mutation. Moreover, the frequency of juxtapapillary RH in patients with truncation mutation was the lowest among the three mutants (26). Dollfus et al. found that patients with complete deletion were associated with a higher incidence of RH, while Michael Reich et al. suggested that patients with truncation mutations had a higher RH incidence (4, 7). Furthermore, this genotype-phenotype association appears to differ in different ethnic groups (27, 28). These results make the genotype-phenotype of ocular VHL disease quite controversial, possibly because the studies were conducted in groups of different race, age and sample size, which also suggests that environmental factors may be involved in the regulation of RH phenotype. Pradeep Mettu et al. further studied the relationship between missense mutations and VHL-associated RH. Their results showed that almost all missense mutations (98.5%) were located in the α or β domain of VHL protein, among which patients with α domain mutation had a higher RH incidence. Moreover, patients with α domain mutation were more likely to have juxtapapillary RH, while β domain mutation was associated with high occurrence of periphery RH (12).

In our case, the patient was detected with a heterozygous missense mutation(c.233A>G) in the VHL gene, leading to an amino acid type change in the VHL protein sequence(p.Asn78Ser) which is located in the β domain. We found that the patient presented mainly with peripheral RH, which was consistent with Pradeep Mettu’s results. In fact, our case is not the first to report that a missense mutation at this locus of VHL gene leads to the pathogenesis of VHL. The first case was reported by F. Chen et al. in 1995, and several other cases were reported in German, Polish, Japanese and Chinese families respectively, indicating that this mutation is relatively common in different races. All of the patients presented with type 1 VHL(without PCC) except one case presenting with type 2 VHL(with PCC), and most of them were associated with RH or CNSH (29–33). Therefore, we believe that VHL patients with c.233A>G missense mutation mainly present type 1 VHL characterized by hemangioma.

### Treatment and follow-up

Clinical management of VHL should be multidisciplinary and comprehensive because of its feature of multi-organ involvement. Here we focus on the treatment of VHL-associated RH. Currently, the selection of therapeutic options for VHL-associated RH is mainly based on the location and size of the lesion and whether there is serious vision-threatening complication such as vitreous hemorrhage, epiretinal membrane or retinal detachment (3, 34). For peripheral RH without the above complications and with a small lesion diameter (usually less than 4.5mm), ablation therapy is mainly used in clinical practice, including laser photocoagulation, cryotherapy, radiotherapy, photodynamic therapy and trans-pupil warm therapy, etc. Among them, laser photocoagulation is relatively common-used, especially for lesions with a diameter of less than 1.5mm, which can often play a very good ablative effect (34–36). In our case, multiple small peripheral RH lesions in the patient’s left eye were treated with direct argon ion laser photocoagulation(532 nm wavelength) of the tumor and its surroundings. Postoperative fundus photography showed good degeneration of the tumor and feeding vessels without retinal hemorrhage or other adverse reactions, indicating the advantages of laser therapy. Cryotherapy has been used to treat lesions 1.5-4.5mm in diameter, but its use is decreasing due to the potential for uncontrollable acute retinal exudation and vitreous contracture (3). Radiotherapy, photodynamic therapy and trans-pupil warm therapy are rarely used because of their unstable efficacy (37–41). Considering that RH, as a vasogenic tumor, is associated with hypoxia response, anti-VEGF and anti-HIF therapy provide a new idea for the treatment of VHL-associated RH (10, 15, 42). Unfortunately, it has not achieved satisfactory results, but it is undoubtedly a promising treatment and deserves further study in the future.

Studies have shown that vitreoretinal surgery is an effective salvage treatment for peripheral RH patients with vitreous hemorrhage, epiretinal membrane, exudative or tractive retinal detachment, or oversized lesions, and can also be used as one of the early treatment methods for small RH lesions with exudative or tractive tendency (3, 17). Surgical options include PPV with ablation therapy,PPV with feeding vessel ligation or PPV with lesion resection, which are still controversial in application at present. In order to avoid vitreous hemorrhage and proliferative vitreoretinopathy, attention should be paid to effectively close the feeding and draining vessels and complete resection of the posterior hyaloid and epiretinal membranes. In addition to hemorrhage and proliferative vitreoretinopathy, postoperative complications include cataract, neovascular glaucoma, retinal detachment, and iatrogenic retinal rupture. In our case, due to the large RH lesion in the patient’s right eye with severe hyperplasia and tractive retinal detachment, we performed PPV, lesion resection, endolaser photocoagulation and silicone oil tamponade. The peripheral location of the tumors makes them easier to remove surgically with minimal visual sequelae. And the 23G PPV procedure ensured minimal iatrogenic injury. Postoperative follow-up showed that the patient had flat retina with no residual lesions, good visual recovery, and no reoccurrence of RH or retinal detachment, suggesting good surgical efficacy. In fact, there have been studies comparing the outcomes and postoperative complications of PPV combined with ablation/ligation or lesion resection. In the study of Gaudric et al., a preliminary comparison found that the visual recovery of patients after ablation was better than that after resection while there seemed to be no difference in reoccurrence of RH and retinal detachment between the two procedures (43). Krzystolik et al. reported that resection was more likely to cause RH recurrence than ablation/ligation (44). Others chose between the two procedures primarily based on their clinical experience, with good recovery but high incidence of postoperative complications seen in both procedures (45–47). In conclusion, vitreoretinal surgery is an effective method for the treatment of severe VHL-associated RH with retinal detachment, but postoperative complications are prone to occur.

For juxtapapillary RH, although active laser treatment can effectively control the progression of RH lesions, it may lead to poor prognosis of vision and impaired visual field due to damage to the retinal nerve fiber layer (3, 48). In contrast, photodynamic therapy, radiotherapy and anti-VEGF therapy have better safety but less satisfactory efficacy. Therefore, in the current situation that there is no ideal treatment for juxtapapillary RH, observation and follow-up can be regarded as a better strategy, especially for those with no obvious exudation. If the lesion progresses rapidly with significant exudation and impaired vision, laser photocoagulation may be considered as appropriate. And vitreoretinal surgery remains the primary consideration in juxtapapillary RH patients with retinal detachment.

For the management of VHL-associated RH, one of the core principles to be followed is early detection of lesions and timely treatment, which requires regular follow-up of patients. All VHL patients and their at-risk relatives should undergo annual ophthalmic examinations starting in childhood, including visual acuity examination, fundus examination, fundus photography, FA, etc. by experienced ophthalmologist (5, 6). For patients with VHL-associated RH who have received previous treatment, especially vitreoretinal surgery, our experience is to conduct ophthalmic follow-up at 2 weeks, 1 month, 2 months, 3 months, 6 months, and 1 year after surgery to evaluate treatment outcomes and disease changes. Follow-up should include visual field examination, intraocular pressure measurement, and anterior segment examination in addition to the above annual screening items to assess postoperative complications. Although VHL-associated RH can have a great impact on patients’ vision, CNSH and RCC, as the primary lethal factors of VHL, are the biggest threats to patients’ health (6). Therefore, ophthalmologists should not only pay attention to the diagnosis and treatment of ocular complications of VHL, but also to patients’ general condition and other organ involvement. The death of the patient’s son in our case taught us a painful lesson about the critical importance of screening for VHL complications outside the eye. For screening and follow-up of other organ lesions, please refer to other literature (5, 6).

## Conclusion

VHL is a rare tumor syndrome with multiple organ involvement and complex genotype-phenotype association. The pathogenesis involves inactivation of VHL tumor suppressor genes in accordance with two-hit hypothesis. The clinical manifestations include RH, CNSH, RCC, PCC and pancreatic neoplasms, among which RH is the most prominent. Ophthalmologic examination such as fundus examination and fundus angiography together with genetic testing are of great importance in the diagnosis of VHL-associated RH. Ophthalmic treatment includes ablation therapy(laser photocoagulation, cryotherapy, etc.) and vitreoretinal surgery. The choice of treatment should be based on the size, location and complications of the lesions. Regular follow-up is important for early detection and control of VHL-associated RH. Here we report a case of binocular RH caused by VHL, in which one of the more severe eyes with retinal detachment was treated surgically and the other eye was treated with laser photocoagulation, and the binocular vision recovered well after surgery. It is hoped to provide a new perspective for the clinical management of VHL-associated RH.

## Data availability statement

The datasets presented in this study can be found in online repositories. The names of the repository/repositories and accession number(s) can be found in the article/**Supplementary Material**.

## Ethics statement

The studies involving human participants were reviewed and approved by the Medical Ethics Committee of the First Affiliated Hospital of Hainan Medical University. The patients/participants provided their written informed consent to participate in this study. Written informed consent was obtained from the individual(s) for the publication of any potentially identifiable images or data included in this article.

## Author contributions

XH perform the surgery. YH, WH and XH collected the data, prepared the material. YH and WH prepared the manuscript. All authors commented on previous versions of the manuscript. All authors contributed to the article and approved the submitted version.

## Funding

This study was supported by grants from the National Natural Science Foundation of China (No.: 81860172, XH and No.: 82160199, XH) and Hainan Provincial Natural Science Foundation of China (No.: 821RC1126, XH).

## Acknowledgments

Approval of the Institutional Review Board (IRB). The study was approved by First Affiliated Hospital of Hainan Medical University.

## Conflict of interest

The authors declare that the research was conducted in the absence of any commercial or financial relationships that could be construed as a potential conflict of interest.

## Publisher’s note

All claims expressed in this article are solely those of the authors and do not necessarily represent those of their affiliated organizations, or those of the publisher, the editors and the reviewers. Any product that may be evaluated in this article, or claim that may be made by its manufacturer, is not guaranteed or endorsed by the publisher.
