# Supplementary material for: Retinal hemangioblastoma in a patient with Von Hippel-Lindau disease: A case report and literature review
Source: Front Oncol. 2022 Nov 2;12:963469. doi: 10.3389/fonc.2022.963469 (PMC9667117; doi:10.3389/fonc.2022.963469)
Supplement: Supplementary file 1 [file DataSheet_1.docx]

Supplementary Material

## Supplementary Figures


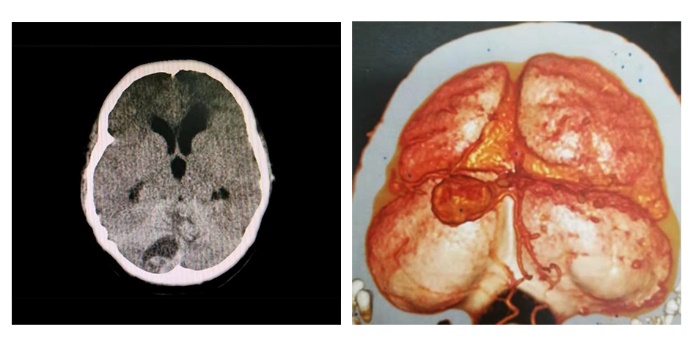


**B**

**A**

Sup.Fig.1. Head CT and CT angiography(CTA) of the patient. Head CT(A) and CTA(B) suggested an hemangioblastoma in the right cerebellar hemisphere.


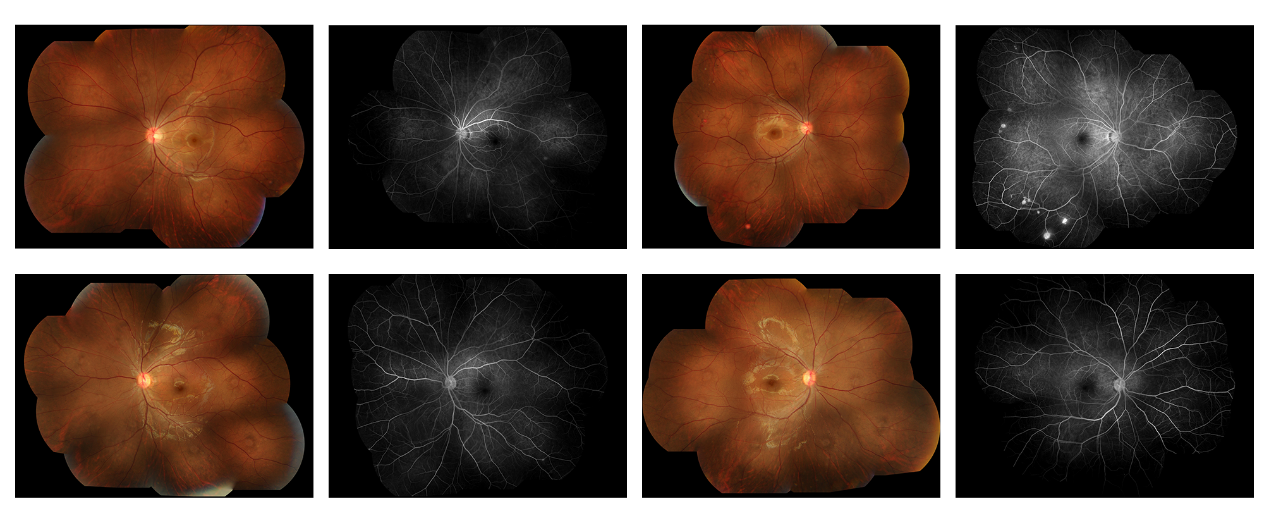


**H**

**G**

**F**

**E**

**C**

**D**

**B**

**A**

Sup.Fig.2. Fundus photography and FA of the patient’s children. Fundus photography(A,C) and FA(B,D) revealed several peripheral RH lesions in both eyes of the patient’s son. Fundus photography(E,G) and FA(F,H) of the patient's daughter were basically normal.


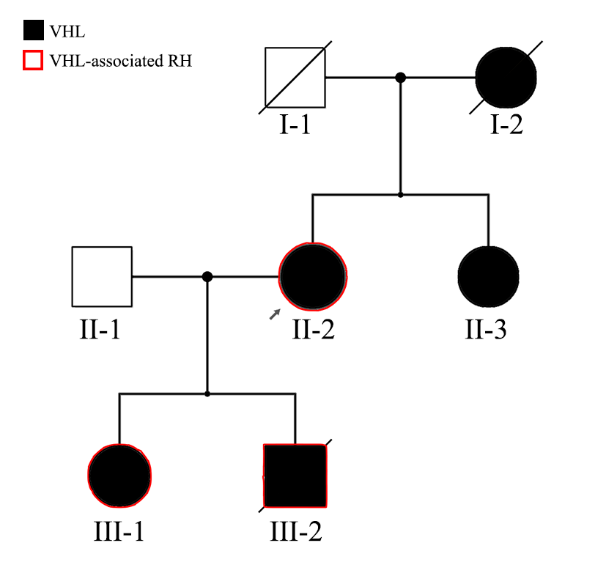


Sup.Fig.3. Genogram of the patient’s family. The genogram was drawn based on the results of sequence analysis and clinical manifestations of VHL-associated RH.


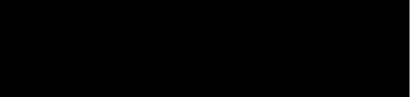


**Surgical treatment of intracranial hemangioma**


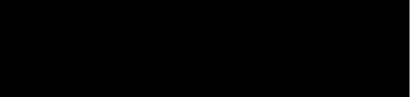


**Multiple cysts in the pancreas and right kidney**

**2019-08-06**

**2019-08-10**

**2019-08-13**

**2019-08-26**

**2019-09-19**

**2019-10-15**

**2019-11-20**


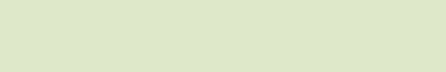


**Vision loss in the right eye**


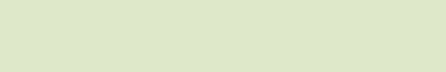


**Ophthalmological examination**


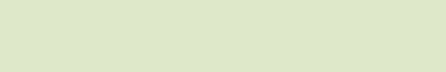


**Multiple RHs with retinal detachment**


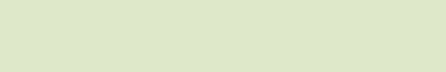


**Fundus photography**


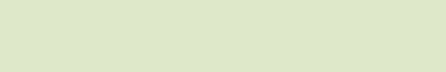


**Suspected VHL**


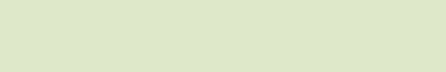


**Fluorescence angiography**


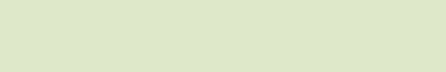


**Optical coherence tomography**


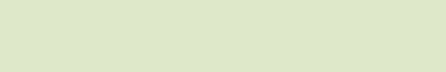


**Confirmed VHL**


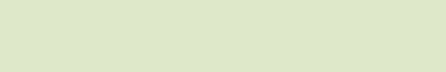


**Genetic sequencing**


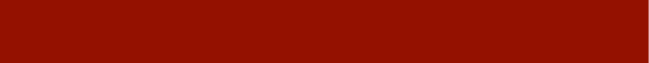


**OS: laser photocoagulation**


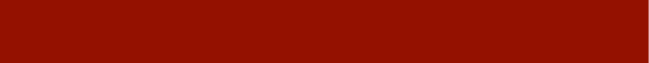


**OD: PPV + lesion resection + endolaser photocoagulation + silicone oil tamponade**


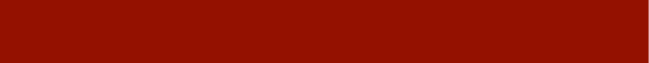


**1^st^ postoperative follow-up**


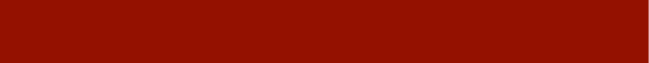


**2^nd^ postoperative follow-up**


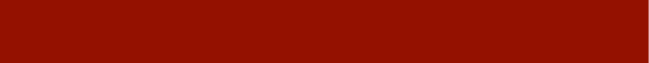


**3^rd^ postoperative follow-up**


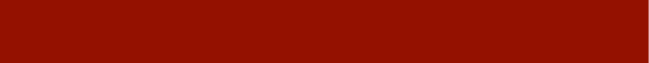


**Silicone oil extraction**


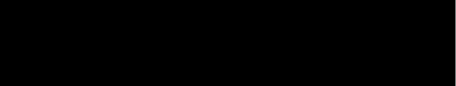


**Visual acuity gradually recovered without recurrence**

Sup.Fig.4. Timeline figure of the case history. Black boxes: relevant past medical history and final resolution of this case. Green boxes: current illness, physical exam, diagnostic evaluations, diagnoses. Red boxes: interventions and follow-ups. Gray arrow: dates. RH: retinal hemangioblastoma. VHL: Von Hippel-Lindau disease. OD: oculus dexter. OS: oculus sinister. PPV: pars plana vitrectomy.
